# A cross-sectional study to assess the level of satisfaction with virtual education in Peruvian medical students

**DOI:** 10.3389/fpubh.2022.1004902

**Published:** 2022-10-05

**Authors:** Pamela Grados-Espinoza, J. Pierre Zila-Velasque, David R. Soriano-Moreno, Kateriny Margot Regalado-Rodríguez, Frank Sosa-Nuñez, William Barzola-Farfán, Jim Gronerth, Lucia Guizado, Christian R. Mejia

**Affiliations:** ^1^Facultad de Medicina Humana, Universidad Nacional Daniel Alcides Carrión, Pasco, Peru; ^2^Red Latinoamericana de Medicina en la Altitud e Investigación, Pasco, Peru; ^3^Unidad de Investigación Clínica y Epidemiológica, Escuela de Medicina, Universidad Peruana Unión, Lima, Peru; ^4^Sociedad Científica de Estudiantes de Medicina de Cajamarca, Cajamarca, Peru; ^5^Facultad de Medicina, Universidad Nacional de Cajamarca, Cajamarca, Peru; ^6^Escuela Profesional de Medicina Humana, Universidad Nacional de San Cristóbal de Huamanga, Ayacucho, Peru; ^7^Sociedad Científica Medico Estudiantil San Cristóbal, Ayacuho, Peru; ^8^Sociedad Científica San Fernando, Lima, Peru; ^9^Facultad de Medicina, Universidad Nacional Mayor de San Marcos, Lima, Peru; ^10^Facultad de Ciencias de la Salud, Escuela de Medicina, Universidad César Vallejo, Trujillo, Peru; ^11^Sociedad Científica de Estudiantes de Medicina de la Universidad César Vallejo, Trujillo, Peru; ^12^Sociedad Científica de estudiantes de Medicina de la Universidad Peruana de Ciencias Aplicadas, Lima, Peru; ^13^Facultad de Medicina Humana, Universidad Peruana de Ciencias Aplicadas, Lima, Peru; ^14^Translational Medicine Research Centre, Universidad Norbert Wiener, Lima, Peru

**Keywords:** epidemiology, evaluation, mental health, Latinos, cross-sectional survey design

## Abstract

**Objectives:**

Education has totally changed in the context of the pandemic. Therefore, the objective of the present study was to evaluate the factors associated with the level of satisfaction with virtual education in Peruvian medical students during COVID-19.

**Methods:**

Analytical and cross-sectional study, based on an online survey of students nationwide. We use previously validated instruments to measure the level of satisfaction and stress (EPP-10-c) of students with virtual education. For the associated factors, adjusted prevalence ratios (PR) were estimated using Poisson regression.

**Results:**

Of the 1,878 students surveyed, the median age was 21 years, 57.8% (1,086) were women, 34.8% (654) had a high level of satisfaction with virtual education and 10.7% (202) presented high levels of stress. The factors associated with a low level of satisfaction were attending the fifth year of study, the partial and non-virtual adaptation of the university to virtual education, and a high level of stress. On the other hand, the factors associated with a high level of satisfaction were the education platform used and the study method used.

**Conclusion:**

Seven out of 10 students presented a low level of satisfaction with virtual education, 1 out of 10 presented a high level of stress. The factors associated with the low level of satisfaction were attending the fifth year of study, the non-virtual and partial adaptation of the university to virtual education, and the high level of stress.

## Introduction

COVID-19 has changed traditional education (1, 2) by interrupting face-to-face education in universities and implementing a virtual modality (3). Virtual education is instruction through technology, where students are physically separated from their teachers (4). However, this modality is not ideal for students' education since, specifically in the case of medicine, it involves many theoretical-practical skills that are acquired in clinical rotations, interacting with patients, or in laboratories (5, 6), with their teachers and colleagues.

Prepandemic, one medical school implemented virtual teaching for some courses, such as Semiology, Clinical Pathology, and Radiology, but not for an entire academic cycle (7). Therefore, the implementation of virtual education is a new experience for most universities. In addition, not all the universities were able to start their activities according to their academic calendar established before the pandemic, and among those that did it, virtual education was not the best (8, 9). This scenario has generated uncertainty and dissatisfaction in student populations in countries such as Nepal, China, and the United Kingdom (3, 10, 11). There are few studies investigating satisfaction in virtual medical education; in one study, good communication between teachers and students was found to be an associated factor (12).

In Peru, the curricular structure is the same in most of the country's medical schools (13), with classroom classes. For now, education virtual will have to play a vital role in the teaching process during pandemic (11) or until it can be controlled. Consequently, knowing the satisfaction of medical students is especially relevant for an adequate implementation in institutions recently adapted to virtual education (14). For this reason, the present study aimed to evaluate factors associated with dissatisfaction with virtual education in Peruvian medical students during COVID-19.

## Methods

### Study design and population

We conducted an analytical cross-sectional study, based on an online, anonymous, self-administered survey for medical students. Twenty-four Peruvian universities with medical schools were evaluated. Convenience sampling was used, because we did not try to extrapolate the results to all medical students in the country or at each university site, but we did try to reach a minimum sample size, calculated at 1,831, to detect a minimum theoretical difference of 3.5% (48.5 vs. 52%), for a power of 85%, a confidence level of 95% and to obtain a single sample (due to the analytical cross-sectional design).

Among the inclusion criteria were considered to be a Peruvian student, to be studying during the pandemic, and acceptance through informed consent to participate in the study.

### Procedure

Participants were enrolled with a survey developed and administered using the Google Forms platform, and it was available for a period of 3 weeks (December 8–29, 2020), when students were about to complete or had completed a year of virtual education in the face of the COVID-19 pandemic so that they could adequately evaluate it after having had the vast majority of their classes in this virtual format. The survey was sent to the contacts of the authors and collaborators of the study through social networks (Facebook, WhatsApp, Telegram, among others) and institutional emails. In addition, it was posted in medical student groups in the aforementioned networks (scientific societies, class groups).

### Questionnaire

The questionnaire consisted of 4 sections: (1) Sociodemographic variables, (2) Experience and methods of virtual education, (3) Student satisfaction and (4) Perceived stress related to the COVID-19 pandemic.

#### Associated sociodemographic factors

We evaluated factors associated with age (years), sex (male or female), region of residence (coast, highlands and jungle), medical school stage (basic sciences, clinical sciences), type of university (public, private), internet access (stable, moderately unstable and very unstable).

#### Experience and methods of virtual education

Educational platform (Google Classroom, Moodle, Schoology, Blackboard, Virtual classroom of the university, Others); virtual classroom (Google Meet, Zoom, GoToMeeting, Blackboard, Microsoft Teams, Others); teaching methodology (resolution of clinical cases, exams resolution such as the National Medical Exam, exam feedback, virtual presentations, virtual simulations, virtual internships, telehealth, journal clubs, discussion of scientific articles, and other forms of teaching), perception of the university's adaptation in regard to virtualization (yes, partially, no).

### Instruments

#### Student dissatisfaction

Was evaluated using the questionnaire developed by Bautista et al. This scale was validated in its version in Spanish in a population of university students and consists of 15 Likert-type questions (Cronbach's alpha coefficient of the original study = 0.92) (15). However, for the present study, items one and fourteen were not evaluated, because they refer to specific education platforms (Microsoft Teams and SMOWL eProctoring, respectively), which are not known by all students. This is why we considered 13 items to be evaluated in the present study. Even so, with these 13 questions the Cronbach's Alpha of our study was 0.91. The scale score ranges from 13 to 65, considering a lower score as a higher dissatisfaction. To define the dissatisfaction variable, the obtained scores were divided into terciles, categorizing the low tercile as dissatisfaction and the two upper terciles as satisfaction with virtual classes (in order to have a cut-off point according to the score of the 13 used questions).

#### Pandemic-related perceived stress scale of COVID-19 (EPP-10-c)

The perceived stress related to the pandemic was evaluated with a 10-item Likert-type scale, each offers 5 response options: never, almost never, occasionally, almost always and always, was previously validated in its Spanish version (Cronbach's alpha = 0.86) (16). A Cronbach's alpha coefficient of 0.81 was obtained for the stress scale we took in our population (which is also within an adequate range). According to the instrument, a cut-off point ≥25 is considered high stress.

### Statistical analysis

The cleaning and coding of the database was performed through the Microsoft Excel program. Subsequently, it was exported and analyzed in the Stata V16.0 program for Windows (Stata Corp, College Station, Texas). For the descriptive analysis of the categorical variables, absolute and relative frequencies were used, and for the numerical variables, measures of central tendency and dispersion were used. In order to find the associated factors, generalized linear models (Poisson family, log link function and models for robust variances) were used to estimate prevalence ratios with their respective 95% confidence intervals and *p*-values. Those statistically significant variables (*p* < 0.05) in the bivariate analysis were included in the multivariate analysis.

### Ethical aspects

The protocol of the present study was evaluated and approved by the institutional ethics committee of Universidad Peruana Unión (Code: 2020-CEUPeU-00047). Virtual consent was obtained from each participant, and the data were anonymous and confidential.

## Results

We were obtained 1,894 responses, 12 did not agree to participate in the study and 4 responses were eliminated because they did not meet the inclusion criteria. Finally, 1,878 responses were considered.

Of the 1,878 students surveyed, the median age was 21 years (interquartile range: 19–23), 57.8% (1,086) were female, 49.5% (931) of the students were from the coast, 55.7% (1,047) were studying basic sciences, the majority of students belonged to private universities 61.7% (1,159), 53.6% had moderately stable internet access, only 34.8% (654) of the students had a high level of satisfaction with virtual education and 10.7% had a high level of stress (Table 1).

**Table 1:** Characteristics of the study population (*n* = 1,878).

**Characteristics**	***N* (%)**
Age (years)^*^	21 (19–23)^*^
**Sex**	
Female	1,086 (57.8)
Male	792 (42.1)
**Place of residence**	
Costa	931 (49.5)
Highlands	861 (45.8)
Jungle	86 (4.5)
**Academic year**	
First year	385 (20.5)
Second year	376 (20.0)
Third year	356 (18.9)
Fourth year	320 (17.0)
Fifth year	218 (11.6)
Sixth year	179 (9.5)
Seventh year	44 (2.3)
**Academic level**	
Basic sciences	1,047 (55.7)
Clinical sciences	831 (44.2)
**University**	
National	719 (38.2)
Particular	1,159 (61.7)
**Educational platform**	
Google classroom	378 (20.1)
Moodle	168 (8.9)
Schoology	2 (0.1)
Blackboard	530 (28.2)
Virtual classroom of the university	682 (36.3)
Others	118 (6.2)
**Virtual classroom**	
Google meet	1,001 (53.3)
Zoom	351 (18.6)
GoTo meeting	6 (0.3)
Blackboard	402 (21.4)
Microsoft Teams	72 (3.8)
Others	46 (2.4)
**Teaching methods used**	
Resolution of clinical cases	1,059 (56.3)
Exam resolution	207 (11.0)
Feedback	309 (16.4)
Virtual presentations	1,337 (71.1)
Virtual simulation	324 (17.2)
Virtual internships	1,054 (56.1)
Telehealth	38 (2.0)
Journal clubs	21 (1.1)
Clinical discussions	463 (24.6)
Others	21 (1.1)
**Internet access**	
Stable	770 (41.0)
Moderately unstable	1,007 (53.6)
Very unstable	101 (5.3)
**Virtual adaptation of the university**	
Yes	459 (24.4)
Partially	1,062 (56.5)
No	357 (19.0)
**Level of satisfaction with virtual education**	
Low	1,224 (65.1)
High	654 (34.8)
**Stress level**	
Low	1,676 (89.2)
High	202 (10.7)

* Median—interquartile range.

**Figure 1:**
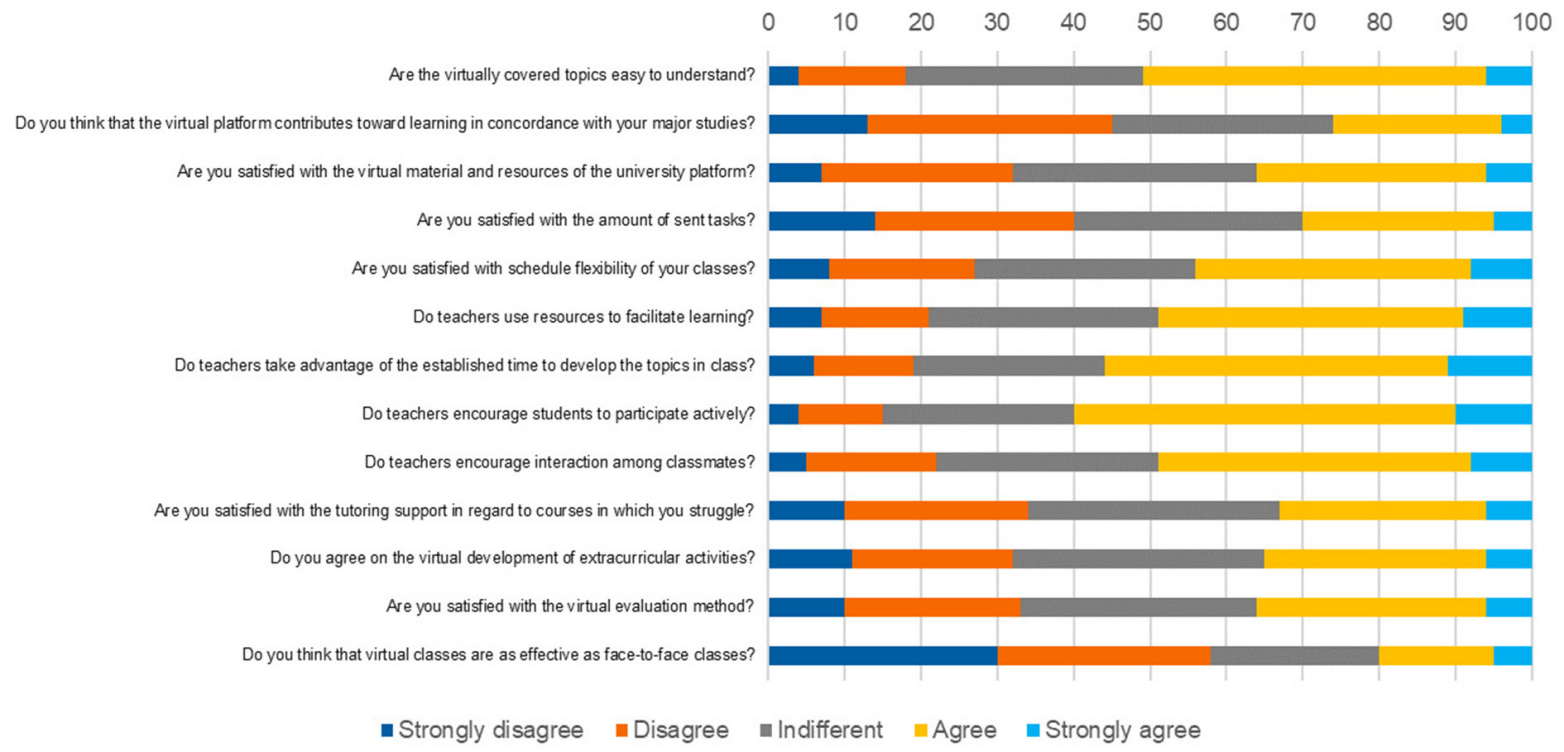
Perception of satisfaction with virtual classes among Peruvian medical students (*n* = 1,878).

Regarding satisfaction with virtual classes, 11% strongly agreed with the fact that teachers take advantage of the established time to develop their topics, 10% though that teachers encourage students to actively participate and 9% that the teachers use resources to facilitate learning (Figure 1).

In relation to the factors associated with the level of satisfaction with virtual education, a significant difference was found with the place of residence (*p* < 0.001), the academic year (*p* < 0.001), the academic level (*p* < 0.001), type of university (*p* < 0.001), the educational platform used (*p* < 0.001), the virtual classroom (*p* < 0.001), if they received feedback as part of their evaluation (*p* < 0.001), simulation (*p* < 0.001), virtual practices (*p* < 0.001), discussion case (*p* < 0.001), internet access (*p* < 0.001), perception of adaptation (*p* < 0.001) and stress level (*p* < 0.001) (Table 2).

**Table 2:** Factors associated with the level of satisfaction with virtual education (bivariate analysis).

**Variables**	**Level of satisfaction**		*P* ^*^
	**Low**	**High**	
	***N* (%)**	***N* (%)**	
**Sex**			0.830
Female	710 (65.3)	376 (34.6)	
Male	514 (64.9)	278 (35.1)	
**Place of residence**			**<0.001**
Costa	568 (61.0)	363 (38.9)	
Highlands	592 (68.7)	269 (31.2)	
Jungle	64 (74.4)	22 (25.5)	
**Academic year**			**<0.001**
First year	213 (55.3)	172 (44.6)	
Second year	235 (62.5)	141 (37.5)	
Third year	250 (70.2)	106 (29.7)	
Fourth year	220 (68.7)	100 (31.2)	
Fifth year	164 (75.2)	54 (24.7)	
Sixth year	122 (68.1)	57 (31.8)	
Seventh year	20 (45.4)	24 (54.5)	
**Academic level**			**<0.001**
Basic sciences	639 (61.0)	408 (38.9)	
Clinical sciences	585 (70.4)	246 (29.6)	
**University**			**<0.001**
National	531 (73.8)	188 (26.1)	
Particular	693 (59.7)	466 (40.2)	
**Educational platform**			**<0.001**
Google classroom	279 (73.8)	99 (26.1)	
Moodle	128 (76.1)	40 (23.8)	
Schoology	0 (0.0)	2 (100.0)	
Blackboard	296 (55.8)	234 (44.1)	
Virtual classroom of the university	445 (65.2)	237 (34.7)	
Others	76 (64.4)	42 (35.5)	
**Virtual classroom**			**<0.001**
Google meet	695 (69.4)	306 (30.5)	
Zoom	230 (65.5)	121 (34.4)	
GoTo meeting	4 (66.6)	2 (33.3)	
Blackboard	206 (51.2)	196 (48.7)	
Microsoft teams	57 (79.1)	15 (20.8)	
Others	32 (69.5)	14 (30.4)	
**Teaching methods used**			
Resolution of clinical cases	675 (63.7)	384 (36.2)	0.137
Exam resolution	120 (57.9)	87 (42.0)	0.021
Feedback	176 (56.9)	133 (43.0)	**0.001**
Virtual presentations	900 (67.3)	437 (32.6)	0.002
Virtual simulation	172 (53.0)	152 (46.9)	**<0.001**
Virtual internships	655 (62.1)	399 (37.8)	**0.002**
Telehealth	18 (47.3)	20 (52.6)	0.020
Journal clubs	10 (47.6)	11 (52.3)	0.089
Clinical discussions	266 (57.4)	197 (42.5)	**<0.001**
Others	16 (76.1)	6 (23.8)	0.287
**Internet access**			**<0.001**
Stable	451 (58.5)	319 (41.4)	
Moderately unstable	689 (68.4)	318 (31.5)	
Very unstable	84 (83.1)	17 (16.8)	
**Virtual adaptation of the university**			**<0.001**
Yes	165 (35.9)	294 (64.0)	
Partially	737 (69.4)	325 (30.6)	
No	322 (90.2)	35 (34.8)	
**Stress level**			**<0.001**
Low	1,057 (63.0)	619 (36.9)	
High	167 (82.6)	35 (17.3)	

* p-value of categorical variables calculated with the Chi Square test. Statistically significant p-values.

In the multivariate analysis (multiple regression) we observed a difference according to the fifth year of study (PR: 0.92; 95% CI: 0.85–0.99; *p* = 0.029), the platform used (schoology) (PR: 1.36; 95% CI: 1.23–1.50; *p* < 0.001), the study method used (virtual simulation and case discussion) PR: 1.04; 95% CI: 1.00–1.08; *p* = 0.021 and PR: 1.04; 95% CI: 1.00–1.07; *p* = 0.023, respectively; the virtual adaptation of education (partially and not) PR: 0.82; 95% CI: 0.79–0.85; *p* < 0.001 and PR: 0.71; 95% CI: 0.68–0.75; *p* < 0.001; and the level of stress (high) PR: 0.89; 95% CI: 0.84–0.93; *p* < 0.001. Adjusted for ten variables (Table 3).

**Table 3:** Factors associated with the level of satisfaction with virtual education (multivariate analysis).

**Variables**	**Level of satisfaction**					
	**Simple regression**			**Multiple regression**		
	***PR***	***IC 95%***	p^**^	***PR***	***IC 95%***	p^**^
**Place of residence**						
Costa	Ref.			Ref.		
Highlands	0.94	0.91–0.97	**0.001**	1.00	0.96–1.03	0.955
Jungle	0.90	0.83–0.97	0.010	0.98	0.90–1.06	0.645
**Academic year**						
First year	Ref.			Ref.		
Second year	0.95	0.90–0.99	**0.044**	0.99	0.94–1.03	0.678
Third year	0.89	0.85–0.94	**<0.001**	0.96	0.91–1.01	0.147
Fourth year	0.90	0.86–0.95	**<0.001**	0.97	0.91–1.03	0.421
Fifth year	0.86	0.81–0.91	**<0.001**	0.92	0.85–0.99	**0.029**
Sixth year	0.91	0.85–0.96	**0.003**	0.99	0.92–1.06	0.856
Seventh year	1.06	0.96–1.18	0.201	1.01	0.98–1.22	0.079
**Academic level**						
Basic sciences	Ref.					
Clinical sciences	0.93	0.90–0.96	**<0.001**	0.98	0.94–1.03	0.591
**University**						
National	Ref.			Ref.		
Particular	1.11	1.22–1.29	**<0.001**	1.00	0.93–1.05	0.858
**Educational platform**						
Google classroom	Ref.			ref.		
Moodle	0.98	0.92–1.04	0.552	0.99	0.93–1.05	0.858
Schoology	1.58	1.53–1.64	**<0.001**	1.36	1.23–1.50	**<0.001**
Blackboard	1.14	1.09–1.19	**<0.001**	1.00	0.94–1.07	0.843
Virtual classroom of the university	1.06	1.02–1.11	**0.003**	1.02	0.97–1.06	0.353
Others	1.07	0.99–1.15	0.053	1.05	0.98–1.13	0.112
**Virtual classroom**						
Google meet	Ref.			Ref.		
Zoom	1.02	0.98–1.05	0.179	1.00	0.95–1.05	0.988
GoTo meeting	1.02	0.76–1.35	0.885	0.92	0.78–1.08	0.339
Blackboard	1.13	1.09–1.18	**<0.001**	1.04	0.99–1.10	0.091
Microsoft teams	0.92	0.85–1.00	0.060	0.94	0.87–1.01	0.100
Others	0.99	0.90–1.10	0.985	1.01	0.91–1.11	0.805
**Teaching methods used**						
Feedback	1.07	1.02–1.12	**0.001**	1.01	0.97–1.05	0.537
Virtual simulation	1.11	1.06–1.15	**<0.001**	1.04	1.00–1.08	**0.021**
Virtual internships	1.05	1.01–1.08	**0.002**	1.01	0.98–1.05	0.224
Clinical discussions	1.07	1.03–1.11	**<0.001**	1.04	1.00–1.07	**0.023**
**Internet access**						
Stable	Ref.			Ref.		
Moderately unstable	0.93	0.90–0.96	**<0.001**	0.98	0.95–1.01	0.344
Very unstable	0.82	0.77–0.88	**<0.001**	0.93	0.87–1.00	0.051
**Virtual adaptation of the university**						
Yes	Ref.			Ref.		
Partially	0.79	0.76–0.82	**<0.001**	0.82	0.79–0.85	**<0.001**
No	0.66	0.64–0.69	**<0.001**	0.71	0.68–0.75	**<0.001**
**Stress level**						
Low	Ref.			Ref.		
High	0.85	0.81–0.89	**<0.001**	0.89	0.84–0.93	**<0.001**

** *P*-values obtained with Generalized Linear Models (GLM), family Poisson, log link function, robust variance. Statistically significant p-values.

## Discussion

The pandemic caused by COVID-19 has had an impact on medical education, these abrupt changes forced the adoption of the virtual teaching modality, which has affected student satisfaction. Our study found that a large percentage of medical students felt that virtual classes were not as effective as face-to-face classes (65.1%). This result was also found in the United Kingdom, where they also stated that family distractions, Internet connection, tutoring schedule, anxiety, lack of space and not being able to learn clinical skills, which are basically acquired in direct contact with the patient, are barriers to effective virtual teaching (11). Another study also found that two out of three Chinese students were not satisfied with the effectiveness of virtual learning (10). Therefore, it could be assumed that students in the clinical sciences phase are mostly those who consider it ineffective, because they need more face-to-face practice and tend to treat virtual education as a complement to traditional methods (10), rather than as a replacement (17). In addition, there is also inadequate implementation of different tools, such as virtual simulation in several universities, which is known to increase the students perception of virtual education as effective (10).

On the other hand, it was found that most of the students were satisfied with the performance of the teachers, because they agreed that their teachers encouraged interaction and active participation among students. In addition, the students believed that their teachers took advantage of the time to develop their topics and used resources to facilitate learning. First, teachers encouraged interaction by generating discussion groups and online case simulations, which have been useful to increase participation (11). Second, they encouraged active student participation as in the United Kingdom, where 60% of students thought that virtual sessions was interactive and found the opportunity to interact through chat messages or talking directly with the professor (11). Third, they made a good use of the time in the development of their subjects because of the advantages provided by virtual education. And finally, teachers employed resources to facilitate learning just as in the United Kingdom and Ireland, where medical educators have used online lectures, videos, virtual simulation, online chat rooms, other technologies and simulation-based teaching modalities (18). Therefore, they applied better learning strategies, as well as, encouraged participation and interaction among students to create a more satisfying learning environment.

With respect to the evaluated teaching methods, it was found that students who received exam feedback showed less dissatisfaction, same result with virtual simulations, virtual internships and clinical discussions. This is due to the fact that interactions such as feedback and answering questions are important factors for student participation during virtual learning; this participation generates a moderate level of satisfaction (10). Feedback is fundamental to medical education because it improves the skills of physicians and students (19). Moreover, it is an effective tool to promote lifelong learning (19, 20) and improve academic performance (21). Having effective feedback after exams with explanation of the key answers and distractors helps students notice their errors and confirm the most appropriate answers. Consequently, this feedback also prepares students for upcoming competitive exams, such as the ENAM in Peru; which will determine their future goals (21).

In virtual education, Internet access is an important factor, and in our study, it was found that students with unstable Internet had a low level of satisfaction. This could occur due to the fact that virtual live classes, exams, presentations, seminars, among others, were frequently interrupted by unstable connection. That happens as a consequence of depending of a smartphone when needing connectivity (due to not having a constant access to a laptop or a desktop computer), which shows a form of insufficient connection. Furthermore, digital devices are too slow for the students' needs and it is necessary to share the devices among members of the family (22). A study in the U.S. reported that problems with Internet connection during virtual education were associated with lower learning competence (22). Another study in the UK found that 22% of students perceived poor Internet connection as a barrier to virtual education (11). It has also been found that students complain more about Internet connection stability than Internet access (23). These findings suggest that in a low-income country like Peru, many students cannot afford a stable and high-speed Internet service, or perhaps they do not have a good Internet signal in their homes, which is a disadvantage and, therefore, makes them feel dissatisfied with virtual education. It is recommended that national education programs and medical schools follow plans to ensure a good internet connection. However, this association is diluted in the multiple regression.

Those students who perceived a greater degree of non-adaptation of their university to virtual education showed a low level of satisfaction. Coping with the new virtual teaching modality may have been difficult due to the challenge of incorporating information technology infrastructures and online platforms in the context of limited or no previous experience with virtual education programs (18). These findings are compatible with a study conducted in the United Kingdom, where only 28% of students reported that their medical school adapted to remote learning (11). In Germany, students had a lower expectation of the implementation of teaching tools, due to a number of factors such as experience with technical problems and remaining wariness caused by changes in conventional teaching (24). On the other hand, inadequate implementation of teacher training could lead to a feeling of incongruence of pedagogical identity with virtual education, which would be related to greater dissatisfaction and frustration in some teachers, and would inevitably generate a negative impact on the student's educational experience (25). Therefore, the gradual and planned incorporation of universities into virtual education is necessary, hence, universities should consider this transition an objective and should be willing to perform constant feedback, in order to improve the development of this new modality.

Regarding the level of stress, it was found that students with a higher level of stress due to the pandemic showed a lower level of satisfaction with virtual education. Pandemics generate mental health burden (26) and, in this context, the perceived stress was due to the impact generated by prevention measures (such as social distancing and quarantines), which restrict people's mobility (16) and limit interpersonal communication (26). In one study, college students were reported to have significantly higher COVID-19 pandemic stress scores (26). In addition, an association between virtual education and stress due to academic, financial, and social difficulties was identified (26). These findings in addition to the dissatisfaction with virtual education found in the students in our study were reinforced by the demand for face-to-face practice. This practice involves the performance of clinical procedures, for which virtual education students may be at a disadvantage due to the lost opportunity to improve these essential clinical skills (27, 28). It is known that virtual education does not meet the necessary requirements to develop all the skills that should be learnt, for example, when evaluating a patient, how to measure their vital signs and symptoms. Therefore, it is necessary to raise substantial issues in order to improve the learning experience and the professional development of medical students.

### Limitations and strengths

Due to not having probability sampling, despite having a fairly adequate sample to find associations, our results cannot be extrapolated because of not having random sampling. In addition, some medical schools may have been disproportionately represented. However, the results were importantly found during the pandemic, where Peru has been the most affected country in the world due to COVID-19, which should serve as a baseline study, so that the universities themselves can evaluate their populations and seek to improve their satisfaction (through the associated factors found in this study).

## Conclusion

In conclusion, 7 out of 10 students presented a low level of satisfaction with virtual education, 1 out of 10 presented a high level of stress. The factors associated with the low level of satisfaction were attending the fifth year of study, the non-virtual and partial adaptation of the university to virtual education, and the high level of stress. It is recommended that medical schools in the country implement an improvement in virtual education taking into account the factors previously described.

## Data availability statement

The original contributions presented in the study are included in the article/supplementary material, further inquiries can be directed to the corresponding author.

## Ethics statement

The protocol of the present study was evaluated and approved by the institutional Ethics Committee of Universidad Peruana Unión (Code: 2020-CEUPeU-00047). Virtual consent was obtained from each participant, and the data were anonymous and confidential. The patients/participants provided their written informed consent to participate in this study.

## Author contributions

PG-E and JZ-V: conceptualization and drafting-revising and editing. PG-E, JZ-V, and CM: methodology. CM: formal analysis and supervision. PG-E, JZ-V, KR-R, FS-N, WB-F, JG, and LG: research. PG-E, CM, PG-E, JZ-V, KR-R, FS-N, WB-F, JG, LG, and CM: data curation. All authors have read and accepted the published version of the manuscript.

## Conflict of interest

The authors declare that the research was conducted in the absence of any commercial or financial relationships that could be construed as a potential conflict of interest.

## Publisher's note

All claims expressed in this article are solely those of the authors and do not necessarily represent those of their affiliated organizations, or those of the publisher, the editors and the reviewers. Any product that may be evaluated in this article, or claim that may be made by its manufacturer, is not guaranteed or endorsed by the publisher.
